# Genetic dissection of novel myopathy models reveals a role of CapZα and Leiomodin 3 during myofibril elongation

**DOI:** 10.1371/journal.pgen.1010066

**Published:** 2022-02-11

**Authors:** Joachim Berger, Silke Berger, Yu Shan G. Mok, Mei Li, Hakan Tarakci, Peter D. Currie

**Affiliations:** 1 Australian Regenerative Medicine Institute, Monash University, Clayton, Australia; 2 Victoria Node, EMBL Australia, Clayton, Australia; The Jackson Laboratory, UNITED STATES

## Abstract

Myofibrils within skeletal muscle are composed of sarcomeres that generate force by contraction when their myosin-rich thick filaments slide past actin-based thin filaments. Although mutations in components of the sarcomere are a major cause of human disease, the highly complex process of sarcomere assembly is not fully understood. Current models of thin filament assembly highlight a central role for filament capping proteins, which can be divided into three protein families, each ascribed with separate roles in thin filament assembly. CapZ proteins have been shown to bind the Z-disc protein α-actinin to form an anchoring complex for thin filaments and actin polymerisation. Subsequent thin filaments extension dynamics are thought to be facilitated by Leiomodins (Lmods) and thin filament assembly is concluded by Tropomodulins (Tmods) that specifically cap the pointed end of thin filaments. To study thin filament assembly *in vivo*, single and compound loss-of-function zebrafish mutants within distinct classes of capping proteins were analysed. The generated *lmod3*- and *capza1b*-deficient zebrafish exhibited aspects of the pathology caused by variations in their human orthologs. Although loss of the analysed main capping proteins of the skeletal muscle, *capza1b*, *capza1a*, *lmod3* and *tmod4*, resulted in sarcomere defects, residual organised sarcomeres were formed within the assessed mutants, indicating that these proteins are not essential for the initial myofibril assembly. Furthermore, detected similarity and location of myofibril defects, apparent at the peripheral ends of myofibres of both Lmod3- and CapZα-deficient mutants, suggest a function in longitudinal myofibril growth for both proteins, which is molecularly distinct to the function of Tmod4.

## Author summary

The force-generating contractile apparatus is a highly organised structure mainly composed of thick and thin filaments of uniform length. Three families of capping proteins are described to play a role in the regulation of thin filament length. Current models suggest that thin filament assembly is initiated by CapZ, extended by Leiomodins (Lmods) and concluded by Tropomodulins (Tmods). To better understand the role of these capping proteins, we analysed single and double loss-of-function zebrafish mutants for these capping proteins. We find that *lmod3*- and *capza1b*-deficient zebrafish model aspects of the human disorders caused by variations in their orthologs. Surprisingly, although pivotal for sarcomere formation, our results reveal that none of the analysed capping proteins, *capza1b*, *capza1a*, *lmod3* and *tmod4*, are absolutely required for thin filament assembly, as suggested by current models. Our study further indicates that the roles of CapZ and Lmod3 are distinct from Tmod4. Both Lmod3- as well as CapZα-deficient mutants feature specific defects at the peripheral ends of muscle cells. We conclude that, in addition to their non-essential role during thin filament assembly, both Lmod3- and CapZα proteins may function in the longitudinal growth of the contractile apparatus.

## Introduction

Sarcomeres, the functional units of the myofibril, are mainly comprised of interdigitated myosin-rich thick and actin-based thin filaments. In skeletal muscle, thin filaments anchor into the sarcomere’s Z-disc and generate contraction force by sliding past thick filaments. Understanding the mechanism by which thin filaments assemble and precisely maintain uniform lengths, while undergoing dynamic turnover, remains an area of active investigation, particularly as deficiencies within this process cause a wide array of muscle weakening diseases including congenital nemaline myopathy [1].

Thin filament assembly and homeostasis is assisted by capping proteins that bind to actin and, by regulating the addition as well as removal of actin monomers, tightly control the length of thin filaments. In the current model, formation of sarcomeric thin filaments starts at the nascent Z-disc (reviewed by [2]), where the barbed end of the filament is located. In short, the chaperonin complex TRiC folds monomeric actin [3], which is passed on to the co-chaperon Bag3 [4]. Bag3 simultaneously interacts with the capping protein CapZ that binds the Z-disc protein α-actinin to form an anchoring complex for thin filaments and actin polymerisation along the nebulin scaffold is initiated [5–8]. Subsequent thin filament dynamics are thought to be facilitated by Leiomodins (Lmods). However, whereas Lmod has been demonstrated to nucleate filaments rather than increasing their elongation rates [9], other *in vitro* studies suggest that Lmod binds to growing ends of actin filaments and accelerates actin polymerization with its three actin-binding sites [10]. Finally, assembly is concluded by Tropomodulins (Tmods) that cap thin filaments specifically at the pointed ends [11,12].

CapZ is a highly conserved heterodimeric complex composed of one α- and one β-subunit. In *Drosophila*, null mutations within the α- and β-subunit encoding orthologs *cpa* and *cpb* cause progressive accumulation of actin filaments in larval imaginal discs [13]. Loss of CAPZB function in human patients was reported to cause hypotonia, cleft palate and micrognathia, which was phenotypically recapitulated in zebrafish mutants harbouring a retroviral insertion into the *capzb* locus [14]. In mammals, the three genes CAPZA1, CAPZA2 and CAPZA3 have been identified to encode for CapZα-subunits [15]. However, a skeletal muscle function has only been discovered for CAPZA2, as patients carrying CAPZA2 variants suffer from hypotonia as well as intellectual disability, speech delay, and seizures [16]. Within the leiomodin protein family, Lmod1 is smooth muscle restricted [17], loss of cardiac Lmod2 induces dilated cardiomyopathy in mice [18] and mutations in Lmod3 can cause nemaline myopathy in humans and mice [19–21]. Tmod4, the predominant of two sarcomeric Tmod isoforms [22], antagonizes the nucleation activity of Lmods by limiting the exchange of actin monomers and caps thin filaments at their pointed ends [12,23,24]. However, conflicting results were obtained using *Xenopus* rescue experiments indicating that the functions of *lmod3* and *tmod4* might be redundant *in vivo* [25].

To better understand the assembly of thin filaments and the associated diseases, we have analysed the skeletal muscle capping proteins within live zebrafish. Loss of *lmod3* function in zebrafish led to weakening of the trunk and head musculature with α-actinin- and actin-positive aggregate formation, which resembles aspects of human nemaline myopathy associated with loss of *LMOD3* [19]. Genetic analysis of *tmod4*;*lmod3* compound mutants demonstrated the distinct roles of both genes for the myofibril *in vivo*. Importantly, all analysed mutants for *lmod3*, *tmod4*, *capza1a*, and *capza1b* surprisingly feature residual muscle striation, indicating that their role during initial myofibril assembly might not be essential as suggested by current models. Furthermore, the surprisingly similar myofibril phenotype of *lmod3*- and *capza1b-*deficient zebrafish indicates that both genes might act within a similar process. The organised array of muscle fibres into myotomes enabled the discovery that the myofibril defects within *lmod3*- and *capza1b*-deficient zebrafish were specifically at the peripheral ends of myofibres. Hence, phenotypic analysis of *lmod3* and *capz1b* mutants suggests that Lmod3 and CapZ might play a role in the longitudinal extension of the myofibril at peripheral myofibre ends.

## Results

### Loss of Leiomodin 3 in *lmod3*^*sa13018*^ leads to compromised muscle integrity

**Fig 1:**
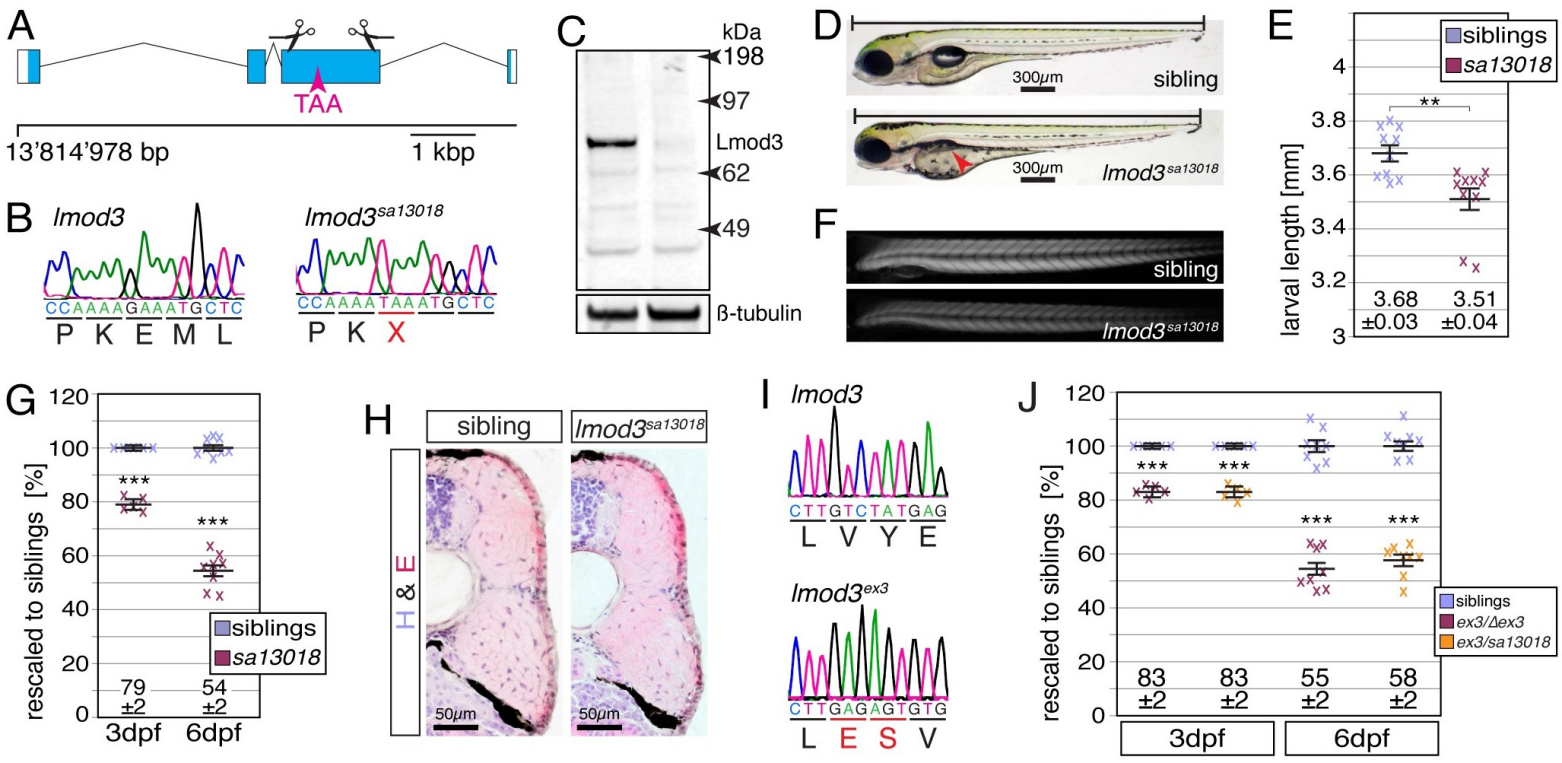
Loss of *lmod3* compromises muscle integrity. (A) The mutant line *lmod3*^*sa13018*^ harboured a premature stop codon within exon 3 of *lmod3*, the start codon of which locates at 13’814’978 bp on chromosome 23. Scissors mark single gRNA targets and scale is 1 kbp. (B) The codon GAA encoding for E338 was changed to the nonsense mutation TAA within *lmod3*^*sa13018*^. (C) Western blot analysis with antibodies against human LMOD3 detected Lmod3 epitope (76 kDa predicted) in siblings (left lane), but not in *lmod3*^*sa13018*^ homozygotes (right lane). ß-tubulin was used as loading control. (D) In contrast to siblings, the swimming bladder (arrowhead) of 4-dpf-old *lmod3*^*sa13018*^ homozygotes was not inflated. The bar indicates larval length without the caudal fin. (E) Whereas 4-dpf-old siblings were 3.68 ± 0.03 mm long, the larval length of *lmod3*^*sa13018*^ homozygotes was with 3.51 ± 0.04 mm significantly shorter (n = 10 larvae). (F) Under polarised light, the muscle of *lmod3*^*sa13018*^ homozygotes appeared darker compared to their siblings due to a reduction in birefringence. (G) After rescaling to siblings, the birefringence of *lmod3*^*sa13018*^ homozygotes was significantly reduced to 79 ± 2% compared to their 3-dpf-old siblings. Crosses represent averaged birefringence of clutches with a minimum of 5 larvae per genotype (n = 5 clutches). At 6 dpf, the birefringence of *lmod3*^*sa13018*^ homozygotes remained significantly reduced to 54 ± 2% of their siblings (n = 8 individual larvae). (H) Signs of fibrosis were absent on cross sections of 6-dpf-old *lmod3*^*sa13018*^ siblings and homozygotes stained with hematoxylin and eosin (H&E). (I) *lmod3*^*ex3*^ harboured a deletion of 1385 bp and insertion of 5 bp in exon 3 of *lmod3*. (J) After rescaling to siblings, the birefringence reduction of *lmod3*^*ex3*^ homozygotes was 83 ± 2% at 3 dpf and 55 ± 2% at 6 dpf. Similarly, *lmod3*^*ex3/sa13018*^ compound heterozygotes had a highly significant reduction in birefringence to 83 ± 2% at 3 dpf and to 58 ± 2% at 6 dpf. At 3 dpf, crosses represent averaged birefringence of clutches with a minimum of 5 larvae per genotype (n = 5 clutches) and individual larvae at 6 dpf (n = 8 larvae). Data are presented as mean ± SEM; ** P < 0.01 and *** P < 0.001 were calculated by Student’s t-test. Scale bar sizes are indicated.

To study *lmod3* deficiency in live zebrafish, the mutant allele *lmod3*^*sa13018*^ from the Sanger Mutagenesis Project was analysed. After out-crossing of *lmod3*^*sa13018*^ to reduce background mutations, sequencing of the *lmod3* locus confirmed a nonsense mutation within exon 3 of *lmod3* (Fig 1A and 1B). Western blot analysis using antibodies raised against human LMOD3 detected the Lmod3 epitope in siblings but not in *lmod3*^*sa13018*^ homozygotes, indicating loss of Lmod3 protein within *lmod3*^*sa13018*^ (Fig 1C). Whereas significantly shorter in length, *lmod3*^*sa13018*^ mutants appeared overall unremarkable at 4 days post fertilisation (dpf) compared to their siblings (Fig 1D and 1E). However, in contrast to their siblings, the swimming bladder of *lmod3*^*sa13018*^ homozygotes was not inflated, indicating a potential defect in locomotion behaviour. To quantify the amount of myofibril within muscle fibres, the birefringence of *lmod3*^*sa13018*^ was analysed. The pseudocrystalline order of muscle sarcomeres gives the myofibril birefringent properties, so that under polarized light the myofibril appears bright in an otherwise dark environment. Under automated and therefore unbiased conditions, the amount of myofibril is directly proportional to the brightness of the myofibril [26]. In contrast to dystrophic muscle characterised by a scattered pattern of birefringence due to detaching and degenerating myofibres [27], the birefringence of *lmod3*^*sa13018*^ mutants appeared uniform (Fig 1F). However, compared to their siblings, the birefringence of *lmod3*^*sa13018*^ homozygotes was significantly reduced at 3 dpf as well as 6 dpf (Fig 1G), demonstrating that the amount of organised myofibril was diminished in leimodin3-deficient mutants; a finding consistent with nemaline myopathy patients suffering from muscle weakness [19]. Consistent with neonatal death of patients with *LMOD3*-associated nemaline myopathy [19], *lmod3*^*sa13018*^ homozygotes died prematurely before 12 dpf. Further pathological assessment of the musculature of *lmod3*^*sa13018*^ with cross sections stained with haematoxylin and eosin (H&E) revealed that 6-dpf-old siblings were overall comparable to *lmod3*^*sa13018*^ homozygotes and neither fibrosis nor necrotic fibres were detected (Fig 1H). The non-dystrophic appearance of *lmod3*^*sa13018*^ homozygotes was in accordance with the unaltered location of the dmd-GFP fusion protein expressed by *Gt(dmd-Citrine)*^*ct90a*^ [28] (S1A Fig). The absence of dystrophic signs within the muscle is consistent with the overall unremarkable appearance of *lmod3*^*sa13018*^ mutants under bright light and their uniform reduction in birefringence.

To confirm that the muscle phenotype of *lmod3*^*sa13018*^ mutants was caused by loss of Lmod3 protein, the CRISPR/Cas9 technology was utilized to independently generate *lmod3* mutants. Two single guidance RNAs were designed targeting exon 3 of *lmod3* and co-injected with Cas9 into zebrafish wildtype (WT) eggs (Fig 1A). After germline transmission, a mutant allele was identified that harboured a deletion of 1385 base pairs (bp) and an insertion of 5 bp in exon 3 (Fig 1I). Thereby the mutant line designated *lmod3*^*ex3*^ featured removal of 69% of the protein coding region of *lmod3*. Birefringence analysis revealed that the birefringence of *lmod3*^*ex3*^ homozygotes was significantly reduced compared to their siblings, at 3 dpf to 83 ± 2% and at 6 dpf to 55 ± 2% (Fig 1J). Importantly, the birefringence of *lmod3*^*ex3/sa13018*^ compound heterozygotes was also significantly reduced to 83 ± 2% at 3 dpf and 58 ± 2% at 6 dpf, which was comparable to single *lmod3*^*sa13018/sa13018*^ and *lmod3*^*ex3/ex3*^ homozygotes. This non-complementation of *lmod3*^*ex3*^ and *lmod3*^*sa13018*^ mutants confirms that the reduced muscle integrity of both lines was caused by *lmod3* deficiency.

In summary, *lmod3*^*sa13018*^ mutants were characterised by absence of Lmod3 protein. The significant reduction of the amount of myofibril at 3 dpf, together with the absence of dystrophic signs indicated that *lmod3*^*sa13018*^ mutants resembled a myopathy like pathology.

### Muscle weakening is apparent in the head and trunk of *lmod3*-deficient zebrafish

**Fig 2:**
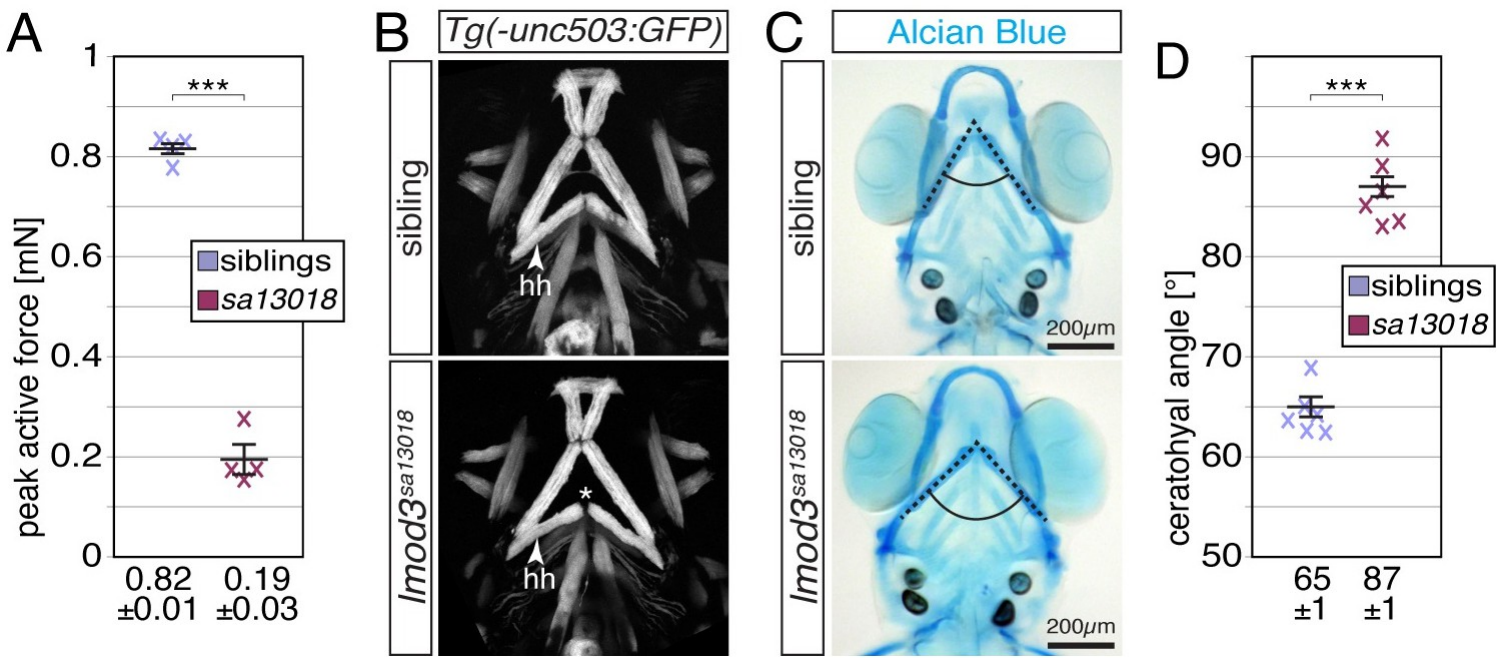
Lmod3-deficiency leads to weakening of the trunk and head musculature. (A) Peak active force of single-twitch contractions generated by 6-dpf-old individual *lmod3*^*sa13018*^ larvae was significantly weaker compared to their siblings. Siblings generated a maximal active force of 0.82 ± 0.01 mN, compared to and 0.19 ± 0.03 mN generated by *lmod3*^*sa13018*^ homozygotes (n = 4). (B) Z-stack projections of ventral views depict the cephalic musculature marked by GFP driven by the *Tg(−503unc*:*GFP)* transgene. In contrast to 4-dpf-old siblings, a gap between the two hyohyoideus (hh) muscles was formed in the lower jaw of *lmod3*^*sa13018*^ homozygotes (asterix). (C) At 7dpf, focus stacks of Alcian Blue cartilage stains revealed a widened jaw within *lmod3*^*sa13018*^ mutants. (D) The angle formed by the ceratohyal cartilages (dotted lines) was significantly wider in *lmod3*^*sa13018*^ homozygotes compared to siblings (87 ± 2° and 65 ± 1°, respectively. n = 6). Data are presented as mean ± SEM; *** P < 0.001 calculated by Student’s t test. Scale bar sizes are 200 μm.

Dysfunctional LMOD3 in nemaline myopathy patients leads to muscle weakness [19]. The reduced amount of myofibril in muscle fibres of *lmod3*^*sa13018*^ mutants detected by birefringence analysis is indicative of muscle weakening also in zebrafish. To further assess the functional deficiency of the skeletal muscle of *lmod3*^*sa13018*^, 6-dpf-old larvae were subjected to force measurements utilising a specialized force transducer. Active force generated by isometric contractions of individual larvae was measured at different lengths to identify the maximal active force at optimum length. Consistent with reduced amount of myofibril, the maximal contractile force generated by *lmod3*^*sa13018*^ homozygotes was with 0.19 ± 0.03 mN significantly less compared to their siblings that were able to generate 0.82 ± 0.01 mN (Fig 2A). As expected from the H&E-stained sections of 4-dpf-old larvae (Fig 1H), the muscle cross-sectional areas (CSA) were similar between 6-dpf-old siblings and *lmod3*^*sa13018*^ homozygotes at comparable anteroposterior levels (0.0318 ± 0.0004 mm^2^ for siblings and 0.032 ± 0.002 mm^2^ for homozygotes). Thereby, the significantly lower active force evident in *lmod3*^*sa13018*^ mutants cannot be attributed to a decrease in muscle size.

Nemaline myopathy patients are characterised by facial and jaw musculature weakness [19]. To analyse whether the head musculature was affected by muscle weakening, *lmod3*^*sa13018*^ mutants were crossed into the transgenic background of *Tg(−503unc*:*GFP)*, which directs GFP fluorescence into the head musculature [29]. Although the head musculature of 4-dpf-old *lmod3*^*sa13018*^ homozygotes appeared anatomically comparable to their siblings, a gap between the two contralateral hyohyoideus muscles was detected (Fig 2B). Similarly, the cartilage formation appeared unchanged in Alcian Blue stained *lmod3*^*sa13018*^ homozygotes (Fig 2C). However, the angle formed by the two ceratohyal cartilage structures was significantly widened in *lmod3*^*sa13018*^ mutants (Fig 2D). As altered muscle strength is known to cause cartilage abnormalities [30], the detected cartilage malformations were indicative of muscle weakness.

Taken together, loss of *lmod3* function leads to weakening of the trunk and head musculature in zebrafish.

### Protein aggregates of *lmod3*-deficient zebrafish resemble nemaline bodies

**Fig 3:**
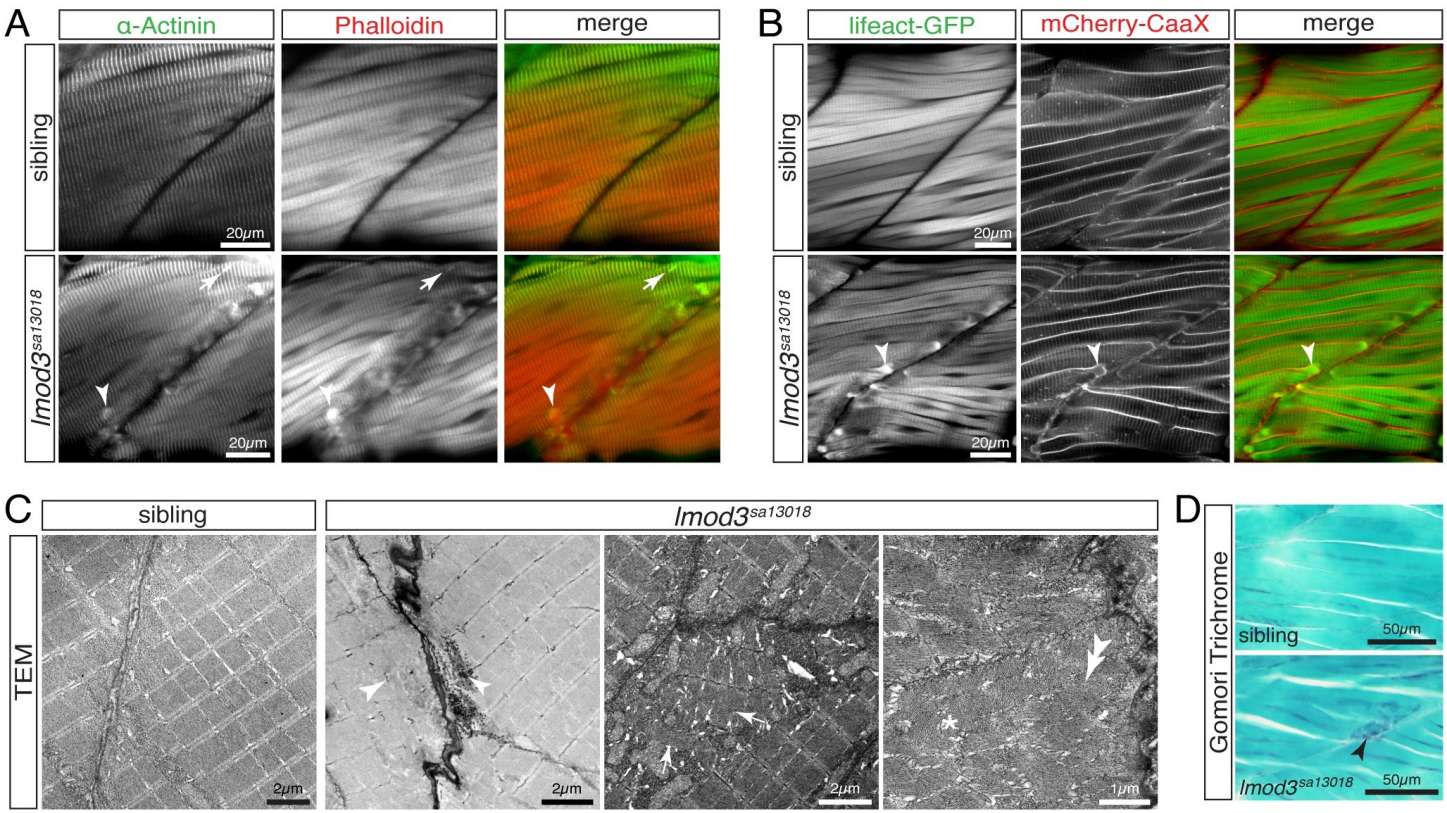
Aggregates within 4-dpf-old *lmod3*^*sa13018*^ are reminiscent of nemaline bodies formed within nemaline myopathy patients. (A) Immunohistochemistry using antibodies against α-Actinin (green) and phalloidin staining (red) marked aggregates located close to vertical myosepta of 4-dpf-old *lmod3*^*sa13018*^ homozygotes. Detected aggregates (arrowhead) were of various sizes and shapes, including rod-shaped structures (arrow). (B) Labelling of the myofibril with Lifeact-GFP (green) and the sarcolemma with mCherry-CaaX (red) confirmed aggregate (arrowhead) formation in *lmod3*^*sa13018*^ homozygotes at 4 dpf, again exclusively at vertical myosepta. (C) At 4 dpf, transmission electron micrographs (TEM) of skeletal muscle from siblings showed the typical myofibril striation and well-aligned sarcomeres. Comparable sarcomeres were also present within *lmod3*^*sa13018*^ homozygotes. However, close to the vertical myosepta, filament deposits reminiscent of fingerprint bodies (star), misaligned sarcomeres (arrows), and electron-dense aggregates of various sizes (arrowheads) were detected. (D) At 5 pdf, Gomori trichrome-stained sagittal sections depicted blue/purple structures close to the vertical myosepta within myofibres of *lmod3*^*sa13018*^ homozygotes. Scale bar sizes are indicated.

A hallmark of dysfunctional LMOD3 in nemaline myopathy patients is the formation of nemaline bodies (rods), aggregates of actin and α-actinin typically marked by Gomori trichrome staining and detected as electron-dense structures on electron micrographs [19]. Immunohistochemistry using antibodies against the Z-disc protein α-actinin revealed aberrant aggregates within *lmod3*^*sa13018*^ myofibres that co-stained for the actin marker phalloidin (Fig 3A). To assess aggregate dynamics within live zebrafish, *lmod3*^*sa13018*^ mutants were crossed into the transgenic background of *Tg(acta1*:*lifeact-GFP)* and *Tg(acta1*:*mCherry-CaaX)* background, in which Lifeact-GFP highlights actin thin filaments and mCherry-CaaX directly marks the sarcolemma [12]. Aggregates were not detected in siblings and 3-dpf-old *lmod3*^*sa13018*^ homozygotes (S1B Fig). However, at 4 dpf actin-positive aggregates were observed by the fluorescence of Lifeact-GFP in *lmod3*^*sa13018*^ homozygotes (Fig 3B), which appeared unchanged at 6 dpf (S1B Fig). To assess if aggregate formation depended on mechanical forces, *lmod3*^*sa13018*^ siblings and homozygotes were kept under anaesthetising conditions from 8 hpf, before muscle contraction commenced. As depicted in the *Tg(acta1*:*lifeact-GFP)* and *Tg(acta1*:*mCherryCaaX)* background, aggregate formation and localisation was unchanged in immobilised *lmod3*^*sa13018*^ homozygotes (S1C Fig), suggesting that aggregate formation was independent from muscle force generation. To further investigate aggregate formation within *lmod3*^*sa13018*^ zebrafish mutants, transmission electron microscopy (TEM) was performed. Organised sarcomeres were found within myofibres of 4-dpf-old siblings and *lmod3*^*sa13018*^ homozygotes, revealing that monomeric actin polymerises to form thin filaments. The average length of organised sarcomeres in *lmod3*^*sa13018*^ homozygotes was 1.522 ± 0.005 μm and comparable to siblings that had a sarcomere length of 1.524 ± 0.008 μm (P = 0.899 calculated by t-test, n = 3). However, *lmod3*^*sa13018*^ myofibres also featured electron-dense structures, sarcomeres of scattered orientation, dispersed filamentous deposits and fingerprint bodies (Fig 3C). Accordingly, sagittal sections stained with Gomori trichrome depicted blue/purple structures typical for nemaline myopathy only in 5-dpf-old *lmod3*^*sa13018*^ homozygotes, not in siblings (Fig 3D). Interestingly, *lmod3*^*sa13018*^ aggregates detected by lifeact-GFP in living transgenic animals as well as by TEM and immunohistochemistry were located at the peripheral ends close to the vertical myosepta, where sarcomeres newly assemble to extend myofibrils longitudinally [31,32]. To assess if the longitudinal myofibril growth could be compromised within *lmod3*^*sa13018*^ mutants, the length of myomeres was measured in 4-dpf-old larvae. Quantification of the length of myomeres between vertical myosepta revealed that myomeres within *lmod3*^*sa13018*^ homozygotes were significantly shorter compared to siblings (S1D Fig), which is in accordance to the significantly shorter larval length of 4-dpf-old *lmod3*^*sa13018*^ mutants. Together with the finding that newly assembled sarcomeres extend myofibrils longitudinally and the impaired sarcomere assembly throughout myofibres, the myomere shortening in *lmod3*-deficient mutants could suggest that Lmod3 plays a role in myofibril extension.

In summary, whereas a reduction in the amount of myofibril was apparent already at 3 dpf, aggregates within *lmod3*^*sa13018*^ appeared later, after the initial myofibril assembly, and featured characteristics reminiscent of nemaline bodies. The simple myotomal arrangement of myofibres within *lmod3*^*sa13018*^ zebrafish mutants revealed the novel finding that aggregates exclusively formed at the peripheral ends of myofibres.

### Loss-of-function mutants of *capza1a* and *capza1b* revealed the redundant function of both genes

**Fig 4:**
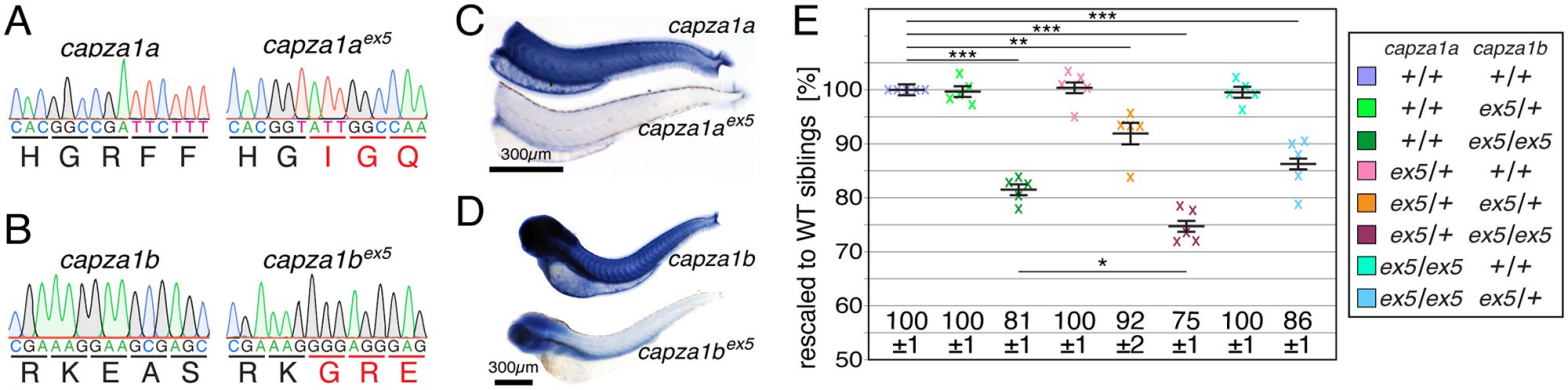
The muscle integrity is compromised in *capza1a*^*ex5*^ but not *capza1b*^*ex5*^ loss-of-function mutants. (A) *capza1a*^*ex5*^ mutants harbour an insertion of 20 bp into exon 5 of *capza1a*, resulting in a frame shift. (B) The mutant allele *capza1b*^*ex5*^ was generated by deletion of 23 bp and insertion of 1 bp into exon 5 of *capza1b*, causing a frame shift. (C) At 3 dpf, *in situ* hybridization showed that the trunk expression of *capza1a* found in siblings was strikingly reduced in *capza1a*^*ex5*^ homozygotes. (D) At 3 dpf, whole-mount *in situ* hybridization showed that the expression of *capza1b* was severely reduced in *capza1b*^*ex5*^ homozygotes compared to siblings. (E) At 3 dpf, the birefringence of single *capza1a*^*ex5/+*^ and *capza1b*^*ex5/+*^ heterozygotes as well as single *capza1a*^*ex5/ex5*^ homozygotes was comparable to WT siblings (*capza1a*^*+/+*^;*capza1b*^*+/+*^, *capza1a*^*ex5/+*^;*capza1b*^*+/+*^, *capza1a*^*+/+*^;*capza1b*^*ex5/+*^ and *capza1a*^*ex5/ex5*^;*capza1b*^*+/+*^ all were 100 ± 1%). However, single *capza1b*^*ex5/ex5*^ homozygotes were significantly reduced in their birefringence and larvae that were additionally heterozygous for *capza1a*^*ex5/ex5*^ were significantly reduced further (*capza1a*^*+/+*^;*capza1b*^*ex5/ex5*^ were 81 ± 1% and *capza1a*^*ex5/+*^;*capza1b*^*ex5/ex5*^ were 75 ± 1%). In addition, compound heterozygotes *capza1a*^*ex5/+*^;*capza1b*^*ex5/ex5*^ and *capza1a*^*ex5/ex5*^ homozygotes that were *capza1b*^*ex5/+*^ heterozygous were also significantly reduced in their birefringence (*capza1a*^*ex5/+*^;*capza1b*^*ex5/+*^ were 92 ± 2% and *capza1a*^*ex5/ex5*^;*capza1b*^*ex5/+*^ were 86 ± 1%). Data are mean ± SEM; * P < 0.05, ** P < 0.01 and *** P < 0.001 calculated by one-way ANOVA with post hoc Tukey’s test; n = 5 clutches. Scale bar sizes are 300 μm.

CapZ heterodimers are composed of α- and β-subunits that cap thin filaments at barbed ends [8]. To study the function of the capZα-subunit in zebrafish, mutants for the two genes *capza1a* and *capza1b* encoding for α-subunits were generated using CRISPR/Cas9 technology. For *capza1a*, a single guidance RNA was designed targeting exon5 and co-injected with Cas9 into WT eggs. After germline transmission, the mutant allele *capza1a*^*ex5*^ was isolated with an insertion of 20 bp into exon 5 of *capza1a* (Fig 4A). Similarly, the mutant allele *capza1b*^*ex5*^, harbouring deletion of 23 bp and simultaneous insertion of 1 bp into exon 5, was isolated (Fig 4B). Exons 5 of both, *capza1a* and *capza1b*, are not subject to alternative splicing, as annotated in the genome assembly (GRCz11) and mutated transcripts within *capza1a*^*ex5*^ and *capza1b*^*ex5*^ were confirmed by RT-PCR (S2A and S2B Fig). To assess if the detected frameshift mutations within *capza1a*^*ex5*^ and *capza1b*^*ex5*^ led to nonsense-mediated decay, *in situ* hybridisation was performed. At 3 dpf, the heads of the offspring from a *capza1a*^*ex5*^ heterozygotes in-cross were used for PCR-based genotyping. Subsequent *in situ* hybridisation with a *capza1a* probe showed a strong signal reduction in the trunks of *capza1a*^*ex5*^ homozygous compared to siblings (Fig 4C). Similarly, *in situ* hybridisation with a *capza1b* probe revealed that the signal intensity was strikingly reduced in *capza1b*^*ex5*^ homozygotes compared to their siblings (Fig 4D). Both *capza1a* and *capza1b* expression patterns resembled previously reported expressions [33] and the strong signal reductions demonstrated the nonsense-mediated decay in *capza1a*^*ex5*^ and *capza1b*^*ex5*^, confirming that both generated lines were loss-of-function mutants. Whereas *capza1a*^*ex5*^ homozygotes reached adulthood, *capza1b*^*ex5*^ homozygotes died at 12 dpf. Birefringence analysis at 3 dpf revealed that the amount of myofibril was significantly reduced in single *capza1b*^*ex5*^ homozygotes compared to siblings, whereas single *capza1a*^*ex5*^ homozygotes did not show a significant reduction in birefringence (Fig 4E). Interestingly, the birefringence of *capza1a*^*ex5/+*^;*capza1b*^*ex5/+*^ compound heterozygotes was also significantly reduced, although the birefringence of single *capza1a*^*ex5*^ or *capza1b*^*ex5*^ heterozygotes was not reduced, indicating that the functions of both genes are redundant. The birefringence of *capza1b*^*ex5*^ homozygotes that harboured an additional *capza1a*^*ex5*^ heterozygous allele (*capza1a*^*ex5/+*^;*capza1b*^*ex5/ex5*^) was further significantly reduced compared to single *capza1b*^*ex5*^ homozygotes. Compound *capza1a*^*ex5*^;*capza1b*^*ex5*^ homozygotes died at 3 dpf, further confirming the redundant function of *capza1a* and *capza1b*.

Taken together, whereas a muscle phenotype was not detected for *capza1a*^*ex5*^ loss-of-function mutants, the muscle integrity of *capza1b*^*ex5*^ loss-of-function mutants was compromised. In addition, the functions of *capza1a* and *capza1b* for muscle integrity were redundant.

### Loss of *capza1b* function leads to muscle weakness specifically within the trunk musculature

**Fig 5:**
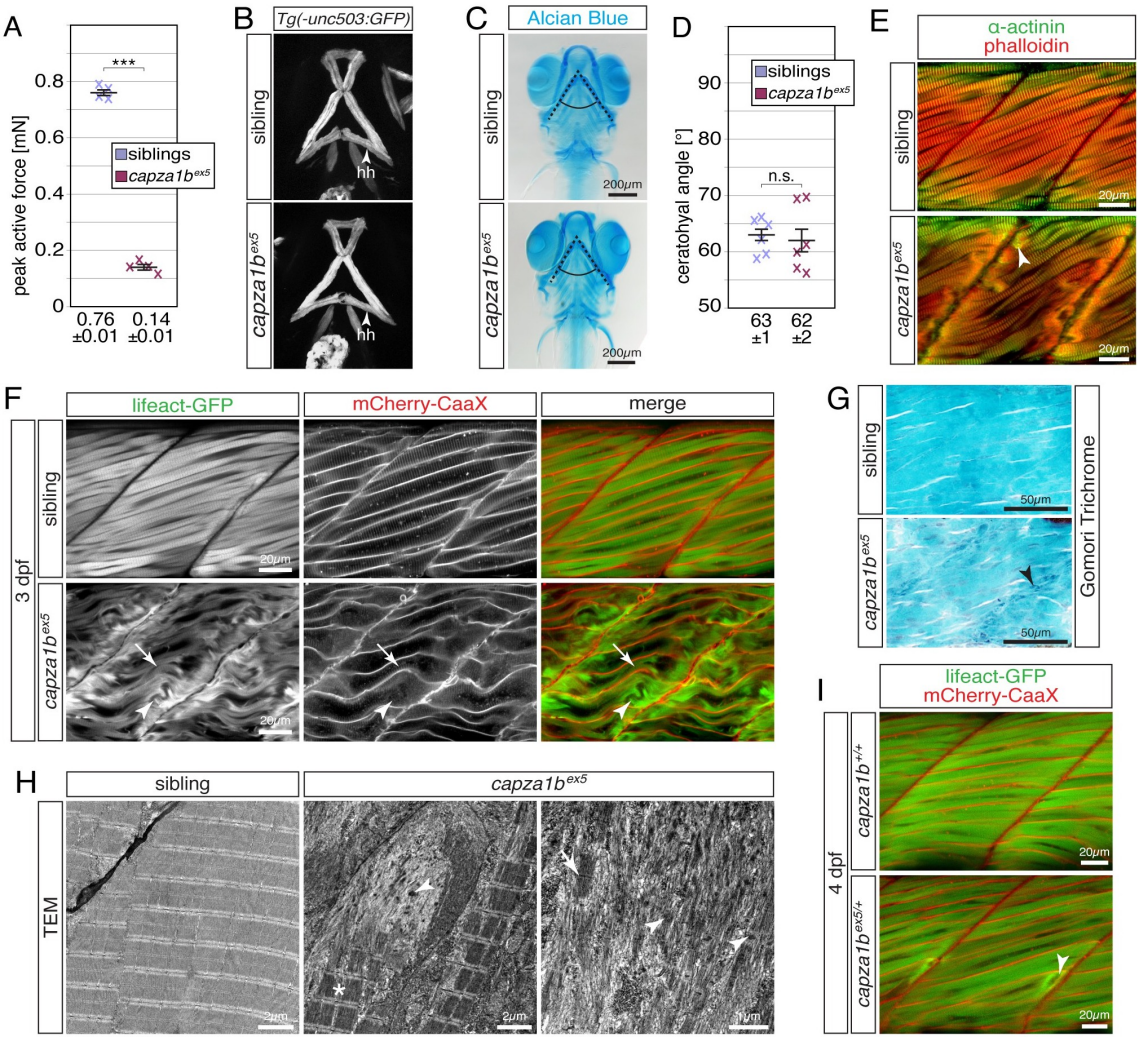
Thin filament deposits and myofibril disruptions lead to muscle weakness within the trunk but not head musculature of *capza1b*^*ex5*^ mutants. (A) Whereas 6-dpf-old siblings were able to generate a peak active force of 0.76 ± 0.01 mN, the force generated by *capza1b*^*ex5*^ homozygotes was significantly reduced to 0.14 ± 0.01 mN (n = 4). (B) GFP fluorescence driven by *Tg(-503unc*:*GFP)* showed that the two hyohyoideus (hh) muscles of *capza1b*^*ex5*^ homozygotes and siblings are comparable at 4 dpf (Z-stack projections of ventral views). (C) Focus stacks of Alcian Blue cartilage stains of *capza1b*^*ex5*^ homozygotes and siblings were comparable at 7 dpf. (D) The ceratohyal angle (dotted lines) of *capza1b*^*ex5*^ homozygotes was with 62 ± 2° not significantly different from the angle of 63 ± 1° formed in siblings (n = 6). (E) Phalloidin (red) and antibodies against α-Actinin (green) marked aggregates (arrowhead) close to the vertical myosepta within 3-dpf-old *capza1b*^*ex5*^ homozygotes but not siblings. (F) Lifeact-GFP (green) highlighted organised myofibril (arrow) in life siblings and *capza1b*^*ex5*^ homozygotes at 3dpf. In addition to the residual striation, *capza1b*^*ex5*^ homozygotes featured myofibril ruptures (arrowhead) as well as aggregates and thin filament deposits at the peripheral myofibre ends. Transgenic mCherry-CaaX (red) was used to mark the sarcolemma and t-tubules. (G) On 3 dpf sagittal sections, Gomori trichrome-staining exposed blue/purple structures (arrowhead) close to the vertical myosepta of *capza1b*^*ex5*^ homozygotes, but not siblings. (H) At 3 dpf, sarcomeres were organised within siblings as revealed by transmission electron micrographs. Organised sarcomeres were also present within *capza1b*^*ex5*^ homozygotes (star). However, sarcomere organisation was lost in *capza1b*^*ex5*^ close to myofibres’ ends and filament deposits, where isolated sarcomeric structures (arrow) and electron-dense aggregates (arrowhead), often with a lattice structure, were found instead. (I) Interestingly, myofibril ruptures were detected in 4-dpf-old *capza1b*^*ex5/+*^ heterozygotes but not WT siblings, demonstrating *capza1b* haploinsufficiency. Data are presented as mean ± SEM; n.s. not significant and *** P < 0.001 calculated by Student’s t test. Scale bar sizes are indicated.

Signs of fibrosis or necrosis were not detected on H&E-stained cross sections of *capza1b*^*ex5*^, suggesting that *capza1b*^*ex5*^ feature a myopathic muscle pathology (S3A Fig). At 3 dpf, *capza1b*^*ex5*^ homozygous larvae were significantly shorter compared to their siblings (S3B and S3C Fig). To further assess the skeletal muscle of *capza1b*^*ex5*^ homozygotes, maximal force generation was quantified. As expected by the reduced muscle integrity, the peak contractile force generated by *capza1b*^*ex5*^ homozygotes is significantly reduced compared to siblings (Fig 5A). Since the CSA of *capza1b*^*ex5*^ siblings and homozygotes was similar (0.0319 ± 0.0003 mm^2^ and 0324 ± 0.0004 mm^2^, respectively), the reduction in peak force of *capza1b*^*ex5*^ is indicative of a muscle weakness. To assess myofibre functionality within the cranial musculature, *capza1b*^*ex5*^ mutants were crossed into the *Tg(−503unc*:*GFP)* background. The contralateral hyohyoideus muscles of 4-dpf-old *capza1b*^*ex5*^ homozygotes were comparable to their siblings and a gap formation between both muscles was not observed (Fig 5B). Accordingly, cartilage malformations were not detected in *capza1b*^*ex5*^ homozygotes stained with Alcian Blue and the ceratohyal angle of mutants and siblings was comparable at 7 dpf (Fig 5C and 5D), indicating that the cranial muscles of *capza1b*^*ex5*^ were not affected. These findings are consistent with patients harbouring variants of the human ortholog CAPZA2 who suffer from hypotonia but not cranial dysmorphisms [16].

Taken together, these results indicate that the trunk but not the cranial musculature of *capza1b*^*ex5*^ is weakened.

### Loss of *capza1b* leads to thin filament deposits and aggregate formation at the peripheral ends of myofibres

To analyse myofibril defects within the trunk musculature of *capza1b*^*ex5*^ mutants, immunohistochemistry using phalloidin and antibodies against α-actinin was performed at 3 dpf. Similar to *lmod3*^*sa13018*^ mutants, accumulations of aberrant α-actinin- and actin-positive aggregates were detected at the peripheral end of *capza1b*^*ex5*^ myofibres (Fig 5E). By utilising the transgenic lines *Tg(acta1*:*lifeact-GFP)* and *Tg(acta1*:*mCherry-CaaX)*, the presence of actin-positive thin filament accumulations was confirmed, and myofibril ruptures were documented within live, 3-dpf-old *capza1b*^*ex5*^ homozygous (Fig 5F). Aggregate formation or localisation was not altered in 3-dpf-old *capza1b*^*ex5*^ homozygotes that were raised under anaesthetic conditions to prevent generation of muscle force (S3D Fig), indicating that mechanical forces did not contribute to aggregate defects. Similar to *lmod3*^*sa13018*^ mutants and in accordance with the significantly shorter larval length (S3C Fig), also the length of myomeres within *capza1b*^*ex5*^ homozygotes was significantly shorter compared to siblings (S3E Fig). According to the detected aggregates, blue/purple structures were exposed by Gomori trichrome staining on sagittal sections of 3-dpf-old *capza1b*^*ex5*^ homozygotes but not siblings (Fig 5G). To further validate these findings, TEM was performed at 3 dpf. Electron micrographs revealed highly organised sarcomeres within siblings and *capza1b*^*ex5*^ homozygotes (Fig 5H). Accordingly, the average sarcomere length of *capza1b*^*ex5*^ homozygotes (1.579 ± 0.005 μm) and siblings (1.58 ± 0.01 μm) was comparable (P = 0.966 calculated by t-test, n = 3). However, close to vertical myosepta of *capza1b*^*ex5*^ myofibres, sarcomere organisation was lost and isolated sarcomeres as well as abundant filament deposits were found instead (Fig 5H). These filament deposits were interspersed with electron-dense aggregates that often showed a lattice structure, which is typical for nemaline bodies [34] (Fig 5H and S3F Fig). Interestingly, at 4 dpf myofibril disruptions and thin filament deposits were detected in some myofibres of *capza1b*^*ex5/+*^ heterozygotes. The requirement of two functional *capza1b* alleles for the myofibril maintenance showed that the levels of Capza1b protein are critical to achieve full functionality of *capza1b*, demonstrating the haploinsufficiency of *capza1b* (Fig 5I).

Taken together, the performed muscle analyses revealed α-actinin- and actin-positive aggregates, as well as thin filament deposits, close to myosepta within *capza1b*^*ex5*^ homozygotes at 3 dpf and slightly later within 4-dpf-old, haploinsufficient *capza1b*^*ex5/+*^ heterozygotes zebrafish.

### Depletion of the α-subunit of CapZ leads to myofibril defects located at the peripheral ends of myofibres

**Fig 6:**
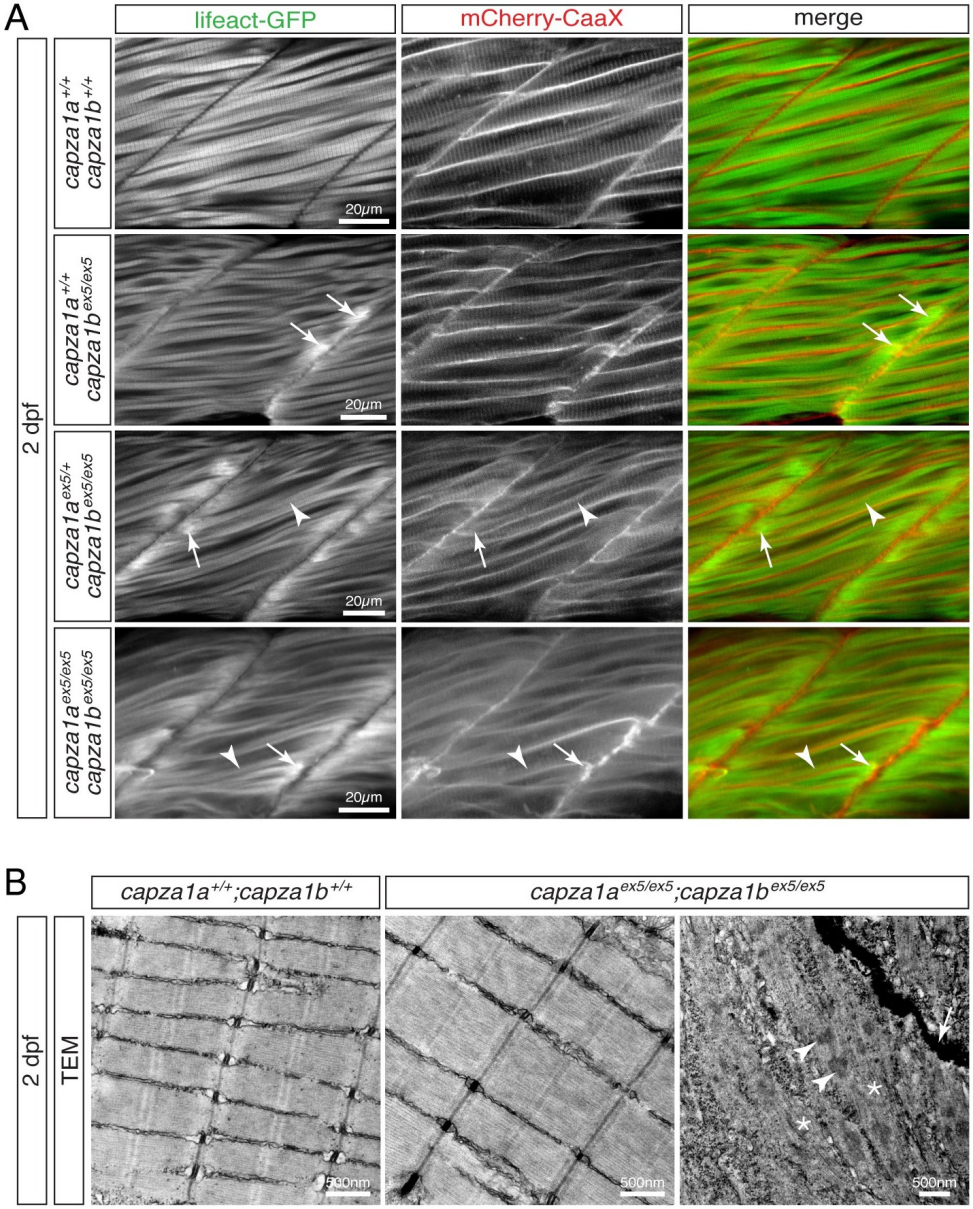
Residual sarcomeres assemble within *capza1a* and *capza1b* depleted zebrafish. (A) At 2 dpf, Lifeact-GFP (green) and mCherry-CaaX (red) highlighted the sarcomere organisation and myofibril striation within muscle fibres of WT siblings. In two out of four analysed *capza1b*^*ex5*^ homozygotes, thin filament deposits (arrows) were detected at the peripheral ends of some fibres. Although myofibril striation (arrowheads) was detected in *capza1b*^*ex5*^ homozygotes that were hetero- or homozygous for *capza1a*^*ex5*^, thin filament deposits (arrows) were frequently located at the peripheral myofiber ends. (B) At 2 dpf, highly organised sarcomeres were found on transmission electron micrographs of siblings and *capza1a*^*ex5*^;*capza1b*^*ex5*^ compound homozygotes. However, electron-dense aggregates with a lattice structure (arrowheads) and filament deposits (star) were present close to the myosepta (arrow) in the compound homozygotes. Scale bar sizes are indicated.

Birefringence analysis of *capza1a*^*ex5*^ and *capza1b*^*ex5*^ single and compound mutants indicated the functional redundancy of both genes. However, any reduction of organised sarcomeres, either by loss of entire myofibres, loss of myofibril or sarcomere disorganisation results in a reduction of birefringence. To analyse loss of the α-subunit of CapZ in more detail, *capza1a*^*ex5*^ and *capza1b*^*ex5*^ mutants were in-crossed in the background of *Tg(acta1*:*lifeact-GFP)* and *Tg(acta1*:*mCherry-CaaX)*. As compound homozygotes die at 3 dpf, the study was performed with 2-dpf-old embryos. Interestingly, thin filament deposits were found in some myofibres within 2 out of 4 analysed *capza1b*^*ex5*^ homozygotes close to peripheral myofiber ends (Fig 6A), indicating that *capza1b*^*ex5*^ myofibrils were initially assembled and then exhibited defects at their peripheral ends at 2 dpf. Furthermore, presence of either one or two *capza1a*^*ex5*^ alleles within *capza1b*^*ex5*^ homozygotes (*capza1a*^*ex5/+*^;*capza1b*^*ex5/ex5*^ or *capza1a*^*ex5/ex5*^;*capza1b*^*ex5/ex5*^) led to abundant thin filament deposits in all analysed 2-dpf-old larvae. Nonetheless, muscle striation was detected in all analysed larvae, including *capza1a*^*ex5/ex5*^;*capza1b*^*ex5/ex5*^ compound homozygotes (Fig 6A). To assess the muscle striation in more detail, TEM was performed at 2 dpf. As expected from the results with live mutants, highly organised sarcomeres that were comparable to siblings were found within *capza1a*^*ex5/ex5*^;*capza1b*^*ex5/ex5*^ compound homozygotes using TEM (Fig 6B) and the sarcomere length was unchanged (1.552 ± 0.006 μm and 1.553 ± 0.007 μm, respectively; P = 0.966, n = 3). However, disorganised filaments were deposited at vertical myosepta in the compound homozygotes. In addition, electron-dense aggregates, which featured a lattice structure typical for nemaline bodies [34], were displayed in *capza1a*^*ex5/ex5*^;*capza1b*^*ex5/ex5*^ compound homozygotes close to the myosepta (Fig 6B and S4 Fig).

In summary, the residual striation detected in *capza1a*^*ex5/ex5*^;*capza1b*^*ex5/ex5*^ compound homozygotes indicates that the α-subunit of CapZ is not essential for the initial myofibril assembly. Instead, the location of the myofibril defects in *capza1a*- and *capza1b*-deficient mutants at the peripheral ends of myofibres indicates that CapZα might have a function during the longitudinal extension of the myofibril.

### The role of *tmod4* during thin filament capping is distinct from the roles of *lmod3* and *capza1b*

**Fig 7:**
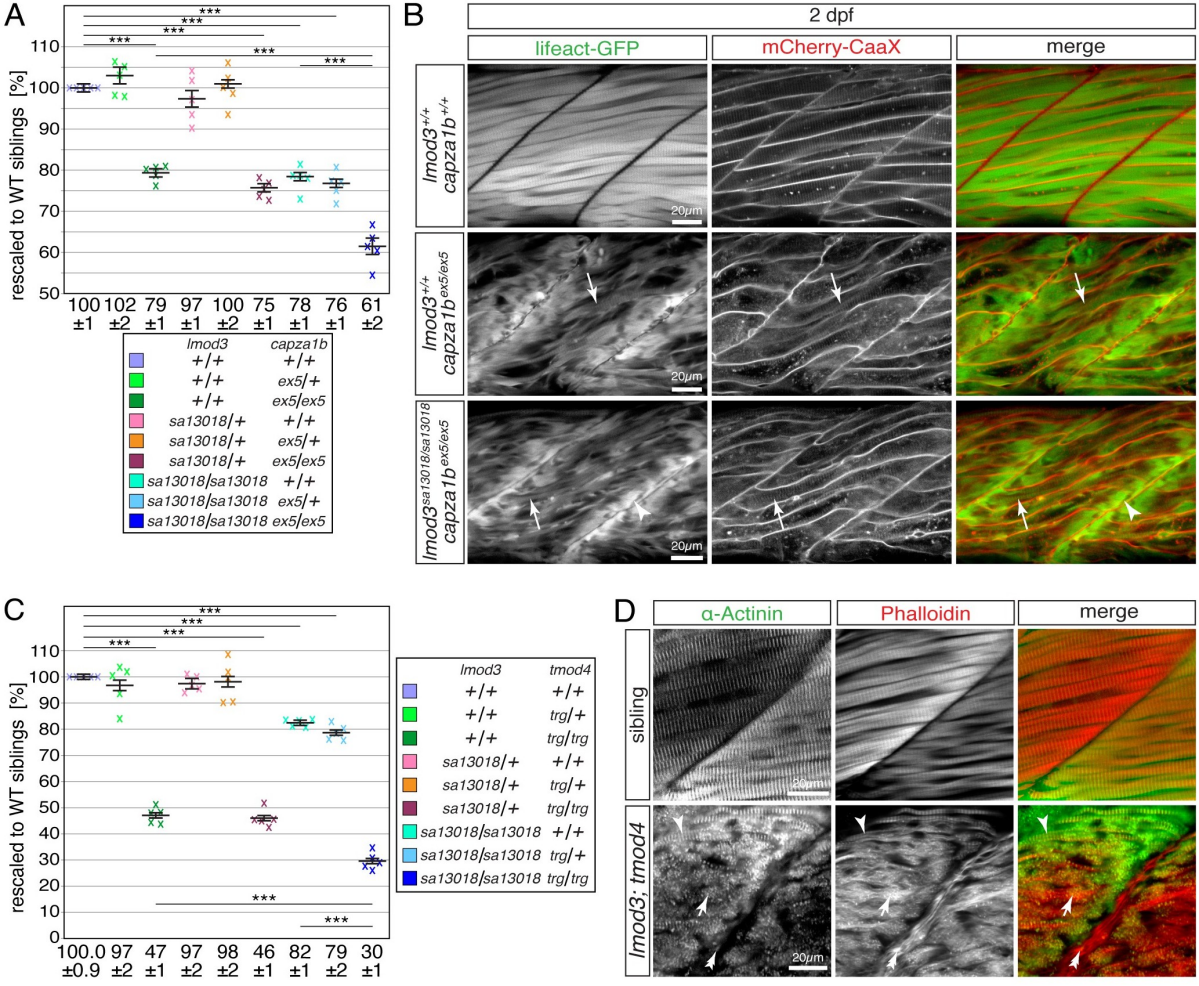
The myofibril defects of *tmod4*-deficient mutants are distinct from the defects of *lmod3-* and *capza1b*-deficient mutants. (A) After rescaling to WT siblings (100 ± 1%), the significant reduction of birefringence of single *lmod3*^*sa13018*^ (78 ± 1%) and *capza1b*^*ex5*^ (79 ± 1%) homozygotes was further reduced in *lmod3*^*sa13018/sa13018*^;*capza1b*^*ex5/ex5*^ compound homozygotes (61 ± 2%). (B) At 4 dpf, *lmod3*^*sa13018*^;*capza1b*^*ex5*^ compound homozygotes featured thin filament deposits at the peripheral ends of myofibres (arrowhead), which were also highlighted in single *capza1b*^*ex5*^ homozygotes by Lifeact-GFP (green) and mCherry-CaaX (red). The strong myofibril striation seen in WT siblings appeared severely reduced in *capza1b*^*ex5*^ homozygotes and was rarely detected in *lmod3*^*sa13018*^;*capza1b*^*ex5*^ compound homozygotes (arrow). (C) After rescaling to WT siblings (100.0 ± 0.9%), the significant reduction of birefringence of single *lmod3*^*sa13018*^ (82 ± 1%) and *tmod4*^*trg*^ homozygotes (47 ± 1%) was further reduced in *lmod3*^*sa13018*^;*tmod4*^*trg*^ compound homozygotes (30 ± 1%). (D) At 3 dpf, the typical myofibril striation was highlighted by antibodies against actinin (green) and actin-labelling with phalloidin (red). In *lmod3*^*sa13018*^;*tmod4*^*trg*^ compound homozygotes striation was rarely seen (arrowhead). Instead, actin- and actinin-positive aggregates were detected throughout myofibres (arrow) as well as actin-positive deposits close to vertical myosepta (double arrow). Data are presented as mean ± SEM; *** P < 0.001 calculated by one-way ANOVA with post hoc Tukey’s test; n = 5 clutches. Scale bar sizes are 20 μm.

Myofibril defects of both *lmod3*- and *capza1b*-deficient mutants manifest at the peripheral ends of myofibres. To compare the muscle phenotype of both mutants in more detail, the muscle integrity of *lmod3*^*sa13018*^ and *capza1b*^*ex5*^ compound mutants was assessed. At 3 dpf, the birefringence of *lmod3*^*sa13018/sa13018*^;*capza1b*^*ex5/ex5*^ compound homozygotes was significantly reduced compared to single *lmod3*^*sa1301*^ or *capza1b*^*ex5*^ homozygotes, indicating that the muscle phenotype of both mutants might be additive and both genes have distinct functions (Fig 7A). However, birefringence is a tool that evaluates the overall muscle integrity and specific myofibril defects are not distinguished. Thus, to further analyse the musculature of these mutants, *lmod3*^*sa13018*^ and *capza1b*^*ex5*^ compound mutants were crossed into the background of *Tg(acta1*:*lifeact-GFP)* and *Tg(acta1*:*mCherry-CaaX)* and analysed at 4 dpf. Robust muscle striation was highlighted by Lifeact-GFP fluorescence in siblings (Fig 7B). In *lmod3*^*sa13018/sa13018*^;*capza1b*^*ex5/ex5*^ compound homozygotes only residual muscle striation was detected and abundant filament deposits characteristic for the single *lmod3*^*sa13018*^ and *capza1b*^*ex5*^ mutants were found instead. Thus, the myofibril characteristics of *lmod3*^*sa13018/sa13018*^;*capza1b*^*ex5/ex5*^ compound homozygotes was similar to the myofibril defects of the single mutants, indicating that although birefringence analysis suggested distinct functions for *lmod3* and *capza1b*, both genes might play a role in a similar process during myofibril assembly.

Similar to *lmod3*^*sa13018*^, the *tmod4*-deficient zebrafish mutant *tmod4*^*trg*^ also features α-actinin- and actin-positive aggregates that could also be detected by TEM [12]. However, in contrast to *lmod3*^*sa13018*^, aggregates and unorganised thin filaments were found dispersed throughout the entire myofibres of *tmod4*^*trg*^ mutants and filament deposits at peripheral ends of myofibers were not detected by transgenic Lifeact-GFP [12]. To assess the functional relation of these two genes, *lmod3*^*sa13018/sa13018*^;*tmod4*^*trg/trg*^ compound mutants were analysed at 3 dpf. Compared to the significant birefringence reduction of the single *lmod3*^*sa13018*^ and *tmod4*^*trg*^ homozygotes, the birefringence of *lmod3*^*sa13018/sa13018*^;*tmod4*^*trg/trg*^ compound homozygotes was significantly reduced further (Fig 7C). To distinguish between the two different types of myofibril defects apparent in single *lmod3*^*sa13018*^ and *tmod4*^*trg*^ mutants, phalloidin staining was combined with antibodies against α-actinin. Two distinct types of myofibril defects were detected within myofibres of *lmod3*^*sa13018/sa13018*^;*tmod4*^*trg/trg*^ compound homozygotes: filament deposits located at the peripheral ends typical of those found in *lmod3*^*sa13018*^ mutants, as well as α-actinin- and actin-positive aggregates dispersed throughout fibres characteristic of those evident in *tmod4*^*trg*^ homozygotes (Fig 7D). Thereby, the myofibril defects that characterise single *lmod3*^*sa13018*^ and *tmod4*^*trg*^ mutants were combined in *lmod3*^*sa13018*^;*tmod4*^*trg*^ compound homozygotes, indicating that *lmod3* and *tmod4* have distinct functions for the myofibril.

Taken together, whereas *lmod3*^*sa13018*^ and *capza1b*^*ex5*^ mutants share a similar myofibril phenotype, the myofibril defects of *lmod3*^*sa13018*^ and *tmod4*^*trg*^ are distinct, indicating that the role of *tmod4* during thin filament capping might be separate from the roles of *lmod3* and *capza1b*.

## Discussion

Thin filament capping is essential to regulate filament length within highly organised sarcomeres. To better understand the role of thin filament capping for the myofibril, the function of the main capping proteins of the skeletal muscle, Capza1a, Capza1b, Lmod3 and Tmod4, were assessed within live zebrafish.

In accordance with the reported thin filament capping functions of CapZ and Lmod3, analyses of *capZα*- and *lmod3*-deficient zebrafish mutants revealed sarcomere defects that were suggestive of a compromised sarcomere assembly. However, alongside the sarcomere defects, residual myofibril striation was detected in all analysed *lmod3*, *tmod4*, *capza1a*, and *capza1b* single as well as compound mutants, which was similarly reported for *capzb*-deficient zebrafish [14]. This unexpected finding indicates that the role of these main capping proteins within skeletal muscle, although pivotal for the overall level of sarcomeres formed, might not be absolutely required, at least for the initial assembly of organised sarcomeres into myofibril. Although the possibility of an early phenotype rescue by the maternal contribution remains, maternally deposited RNA is degraded during the maternal-to-zygotic transition to enable gastrulation [35], well before the first muscle contraction occurs at 17 hpf [36].

Defective sarcomeres within *lmod3*^*sa13018*^ and *capza1b*^*ex5*^ mutants were also reflected by a significantly reduced force generation by both mutants. However, in addition to the significantly reduced amount of organised sarcomeres and significantly shortened myomeres, other factors could contribute to the detected muscle weakness. Whereas reduced CSA, altered location of the myofibril anchoring dystrophin and a reduction in thin filament length were ruled out as likely causes, other possibly contributing factors such as impaired excitation-contraction coupling in conjunction with altered calcium homeostasis or reduced actin and myosin cross bridging dynamics were not assessed to further analyse the functionality of residual sarcomeres.

The parallel alignment of zebrafish myofibres into myotomes revealed that the myofibril defects within *lmod3*- and *capza1b*-deficient zebrafish exclusively located close to the vertical myosepta at the peripheral ends of myofibres. At the initial stage of myofibril assembly, z-bodies of the pre-myofibril align in register to form nascent myofibrils which further develop into mature myofibril [32]. After the initial myofibril formation, the myofibril elongates at its peripheral ends by adding newly assembled sarcomeres in series to facilitate the longitudinal growth of myofibres in fast growing myotomes [31,32]. Hence, the peripheral location of the detected myofibril defects provoked by *lmod3*- and *capza1b*-deficiency suggests that, in addition to the initial phase of myofibril assembly, Lmod3 and CapZ might also have roles during myofibril extension. A potential function during myofibril extension was also supported by the finding that myomeres of *lmod3*- and *capza1b*-deficient mutants were shortened.

To assess the functional relation of Lmod3 to the structurally similar Tmod4, phenotypic analysis of *tmod4*^*trg*^;*lmod3*^*sa13018*^ compound mutants was performed. Both single *lmod3*- and *tmod4*-deficient mutants feature α-actinin- and actin-positive aggregates, but their location within myofibres is distinct with *lmod3*^*sa13018*^ aggregates locating at the peripheral end and *tmod4*^*trg*^ aggregates being dispersed throughout myofibers. Immunohistological analysis revealed that aggregates were located at peripheral ends and dispersed throughout myofibers of *tmod4*;*lmod3* compound mutants and the muscle integrity was further reduced compared to single homozygotes, as expected from an additive effect of two different types of aggregates. The distinct aggregate distribution pattern indicates that both aggregate types originate from defects in distinct processes, thereby indicating that *lmod3* and *tmod4* have different functions during thin filament assembly or maintenance *in vivo*. These results are in agreement with structural and biochemical *in vitro* studies, which suggest that the nucleation function of Lmod3 is distinct from the capping function of the structurally related Tmod4 [24] and do not confirm *in vivo* results from *Xenopus* that suggest redundant functions for *tmod4* and *lmod3* during thin filament assembly [25].

Birefringence analysis of *lmod3*^*sa13018*^;*capza1b*^*ex5*^ compound mutants suggested that *lmod3* and *capza1b* have distinct functions. However, in contrast to *tmod4*^*trg*^ mutants, *lmod3*- and *capza1b*-deficient zebrafish share surprisingly similar phenotypic characteristics within the trunk musculature, although their severity levels differ. Filamentous deposits as well as α-actinin- and actin-positive aggregates exclusively located at the peripheral ends of myofibres within both *lmod3*- and *capza1b*-deficient mutants indicate that both gene functions might contribute to a similar process during thin filament assembly. Thus, the notion that Lmods act together with CapZ in the nucleation process of thin filaments, as suggested for *lmod2* within rat cardiomyocytes [9], is supported rather than their proposed role in an elongation process separate from CapZ [10].

In addition to the reported morpholino model generated by knockdown of *lmod3* [19], *lmod3*^*sa13018*^ mutants were characterised to generate a genetically tractable model for *LMOD3*-deficient nemaline myopathy. Similar to human patients [19], *lmod3* loss-of-function in zebrafish resulted in weakening of the head and trunk musculature. Sarcomere organisation within *lmod3*^*sa13018*^ myofibres was compromised and aberrant α-actinin- and actin-positive aggregates were detected along with electron-dense structures, which resemble nemaline bodies found in *LMOD3*-deficient nemaline myopathy [19]. Nemaline bodies, that clinically define nemaline myopathy, are coloured blue/purple in Gomori trichrome staining and on electron micrographs appear as rod-shaped or ovoid electron-dense structures that are often derived from Z-discs and therefore feature a similar lattice structure and protein content [1,34]. Whereas Gomori trichrome staining indicated the presence specifically of nemaline bodies within *lmod3*^*sa13018*^ myofibres, aggregates detected by TEM did not feature a lattice structure. However, the fingerprint-resembling structure of the filament deposits within *lmod3*^*sa13018*^, as well as their location at peripheral myofiber ends, matched the sub-sarcolemmal location of filamentous fingerprint bodies described for nemaline myopathy patients [20] Thereby, *lmod3*-deficient zebrafish resemble aspects of the clinical symptoms of *LMOD3*-associated nemaline myopathy.

The weakened trunk musculature of *capza1b*-deficient zebrafish reflected the muscle of individuals harbouring *CAPZA2* variants that was reported as hypotonic [16]. A muscle pathology of patients with *CAPZA2* variants was not further described and the reported neurological symptoms could be the basis of the patient’s hypotonia. However, the features of the *capza1b*^*ex5*^ pathology, specifically the Gomori trichrome staining and the lattice-patterned electron-dense structures together with the well-described role of CapZ during thin filament assembly within skeletal muscle, suggest that it might be of interest to explore the muscle symptoms of individuals harbouring CAPZA2 variants in future studies.

In contrast to the weakened trunk musculature of *capza1b*-deficient zebrafish, a weakened cranial musculature as seen in *lmod3*-deficient mutants was not detected, indicating that *lmod3* and *capza1b* have distinct functions within head muscles. However, a cranial defect could also be prevented by a redundant function of both *capza1a* and *capza1b* genes, as the compromised muscle integrity caused by haploinsufficient *capza1b* was aggravated by *capza1a*-deficiency in the trunk and both proteins are 87% identical in their amino acid sequence. CapZα-deficient head muscles were not analysed as head muscles only start developing at 2 dpf and *capza1a*;*capza1b* compound mutants die at 3 dpf. Nonetheless, the functions of zebrafish *lmod3* and *capza1b* seem comparable to their human orthologs *LMOD3* and *CAPZA2*, as patients harbouring *LMOD3* variants are characterised by severe facial and jaw weakness whereas the hypotonic patients harbouring *CAPZA2* variants did not present with facial dysmorphism [16,19].

Collectively, the characterisation of *lmod3*, *tmod4*, *capza1a*, and *capza1b* zebrafish mutants suggests that their functions during the sarcomeric thin filament assembly within skeletal muscle are not absolutely required for the initial assembly of organised myofibril. Furthermore, Lmod3 and CapZ have functions during longitudinal myofibril growth and act distinct from Tmod4. The generated *lmod3*- and *capza1b*-deficient mutants model aspects of the human conditions associated with *LMOD3* and *CAPZA2* variants. In addition, the aggregates of *capza1b*-deficient mutants featured characteristics of nemaline bodies indicating that individuals with *CAPZA2* variants might suffer from nemaline myopathy.

## Material and methods

### Ethics statement

All animal experiments were approved by the Monash University Animal Ethics Committee (ERM22161).

### Generation, maintenance and genotyping of zebrafish lines

Zebrafish were maintained in the TU (Tübingen) zebrafish strain. To raise immobilised larvae, animals were anaesthetised from 8 hpf with 0.01% Tricaine until imaging was concluded. The *lmod3*^*sa13018*^ mutant was obtained from the Zebrafish International Resource Center (Eugene, USA). Mutant lines *lmod3*^*ex3*^, *capza1a*^*ex5*^ and *capza1b*^*ex5*^ were generated by co-injection of Cas9, tracrRNA and crRNA (IDT) into zebrafish eggs as described [3]. The crRNAs targeting 5’-agagctggaacttgtctatgAGG and 5’-tttggtggctctcgcctacgCGG were used for *lmod3*^*ex3*^, 5’-gagggtcaaagaatcggccgTGG for *capza1a*^*ex5*^ and 5’-agcgatcctcaaccgtatgaGGG was used to generate *capza1b*^*ex5*^.

Genotyping of *tmod4*^*trg*^ was described [12]. For *lmod3*^*sa13018*^, the primers lmod3-F (5’-cccaaaacttgctctaggtggc) and lmod3-EcoRI-R (5’-cagggagttgacgtaatcaaggaggaatt) were used to amplify a 184-bp product in a PCR reaction. Subsequent restriction digestion with EcoRI cleaved only the WT amplicon into 155-bp and 29-bp fragments. The CRISPR/Cas9-generated alleles were identified by PCR using the following primer pairs: lmod3-2F (5’-gcagcgtttcagacaggaca) and lmod3-2R (5’-taggccacattgctctgtcg) for *lmod3*^*ex3*^; capZa1a-F (5’-ggggtcgtgggctgaaaagt) and capZa1a-R (5’-tacagcatctctccacggcc) for *capza1a*^*ex5*^; capZa1b-F (5’-gcatggagatctgggtcagg) and capZa1b-R (5’-tgaccgtcaacagttttcccata) for *capza1b*^*ex5*^.

### Quantification of birefringence

The Abrio LS2.2 microscope was utilised to automatically image individual zebrafish larvae in an unbiased way as previously reported [26]. Subsequently, the first 20 somites of imaged larvae were selected and the mean of all grey values of the pixels was measured using the software ImageJ. To enable comparison of the birefringence from different lines, obtained grey values were rescaled to control siblings set to 100%. Siblings were rescaled by multiplying measured grey values (A_1_ to A_n_) of each larva by 100 and dividing the average of all sibling grey values using $\frac{A_{i}\times 100}{\sum_{i=1}^{n}A_{i}/n}$. The grey values (B_1_ to B_n_) of each mutant larva were multiplied by 100 and divided by the average of the measured grey values of the siblings using $\frac{B_{i}\times 100}{\sum_{i=1}^{n}A_{i}/n}$.

For analyses of single mutants, 5 clutches with a minimum of 5 larvae per genotype were assessed for 3-dpf-old larvae and 8 individual larvae were evaluated per genotype for 6-dpf-old larvae. For compound mutants at 3 dpf, 5 clutches with a minimum of 3 larvae per genotype were analysed.

### Statistical analysis

Significance between two groups was determined by Student’s t test and for multiple groups one-way ANOVA with post hoc Tukey’s test was used. Statistical significance was calculated by the software Prism (GraphPad Software). Presented data are mean ± SEM, calculated utilizing error propagation.

### Muscle force measurement

At 6 dpf, larvae were individually mounted at slack length between a force transducer and a puller as described [37]. In short, single twitches were stimulated through electrical pulses of 0.5-ms duration and the generated force was measured. To enable force generation at the optimal larval length (maximal active force), the distance between transducer and puller was increased in between stimulations until larvae reached a length above that giving maximal for active force. Subsequently, all animals were genotyped by PCR as described above.

### Immunohistochemistry, histology and *in situ* hybridisation

Immunohistochemistry and histological stains were performed on 10μm cryofrozen sections according to standard methods. Transcripts of *capza1a* and *capza1b* were detected by *in situ* hybridisation using RNA probes that aligned to the 3’-UTRs. Western blot and Alcian Blue whole mount staining were performed as described [12]. Alexa Fluor-568-conjugated phalloidin (1:1000; Life Technologies A12380) and primary antibodies against α-actinin (1:1000; Sigma A7811) and LMOD3 (1:500; Proteintech 14948-1-AP) were used. Fluorescence images were recorded with an LSM 710 fluorescence confocal microscope (Zeiss, Germany). For electron microscopy, larvae were fixed in 2.5% glutaraldehyde in 0.1 M sodium cacodylate overnight at 4°C, a procedure that might induce alterations in tissue appearances. Electron micrographs of ultrathin sections were taken on a JEM 1400-Plus transmission electron microscope (JEOL, Japan). Unless described otherwise, all experiments were performed with a minimum of three independent biological replicates.

### Sarcomere length measurement

Sarcomere length, from Z-disc to Z-disc, was measured on TEM micrographs using the ImageJ software. Per genotype, the sarcomere length of 3 sarcomeres was measured on 2 TEM micrographs.

## Supporting information

S1 Fig Dynamics of actin-positive aggregates of *lmod3*^*sa13018*^ mutants. (A) At 4 dpf, the dmd-GFP fusion protein expressed by *Gt(dmd-Citrine)*^*ct90a*^ localised at the myotendinous junction in siblings and *lmod3*^*sa13018*^ homozygotes. (B) Actin-positive aggregates were not detected in the background of *Tg(acta1*:*lifeact-GFP)* (green) and *Tg(acta1*:*mCherryCaaX)* (red) at 3dpf; neither in siblings nor *lmod3*^*sa13018*^ homozygotes. However, first signs of disconnected myofibril were apparent (arrowhead). At 6 dpf, aggregates marked by Lifeact-GFP were all localised in *lmod3*^*sa13018*^ at the peripheral end of myofibres. (C) In contrast to 4-dpf-old siblings, aggregates formed and localised at the peripheral end of myofibres also in *lmod3*^*sa13018*^ homozygotes that were raised under anaesthetising conditions to prevent force generation. (D) Whereas the length of rostral myomeres between vertical myosepta was 109 ± 0.9 μm in 4-dpf-old siblings, myomeres within *lmod3*^*sa13018*^ mutants were 97.6 ± 0.7 μm long and significantly shorter in comparison to their siblings (n = 10 larvae with 1 myomere each, P < 0.001). Scale bar sizes are 20 μm.
(TIF)

S2 Fig The mutations of *capza1a*^*ex5*^ and *capza1b*^*ex5*^ are transcribed into mutant transcripts. (A) RT-PCR using RNA isolated from 3-dpf-old *capza1a*^*ex5*^ homozygotes generated a single 220-bp amplicon compared to the 200-bp amplicon generated with RNA obtained from wildtype siblings. The primers capza1a-2F (5’-acatggatcagttcacacctgc-) and capza1a-R (5’- tacagcatctctccacggcc-) were used to amplify *capza1a* cDNA. (B) Whereas the RT-PCR using RNA isolated from 3-dpf-old *capza1b*^*ex5*^ homozygotes resulted in a 151-bp amplicon, a 173-bp amplicon was generated with RNA isolated from wildtype siblings. Amplicon sizes are indicated. PCR with *capza1b* cDNA was performed with capza1b-2F (5’-tggatggcagtgaggagtcg-) and capza1b-2R (5’-acaagcgtctctccaggacc-).
(TIF)

S3 Fig The musculature of *capza1b*^*ex5*^. (A) At 6 dpf, signs of fibrosis or necrosis were absent on H&E-stained cross sections of *capza1b*^*ex5*^ and siblings. (B) At 3 dpf, *capza1b*^*ex5*^ homozygotes appeared shorter compared to their siblings. (C) Measurement of the body length excluding the caudal fin revealed that 3-dpf-old *capza1b*^*ex5*^ homozygotes with a body length of 3.30 ± 0.01 mm were significantly shorter compared to their 3.49 ± 0.01 mm long siblings. Data are presented as mean ± SEM; n = 10 and *** P < 0.001 calculated by Student’s t test. (D) As depicted in the *Tg(acta1*:*lifeact-GFP)* and *Tg(acta1*:*mCherry-CaaX)* background, also 3-dpf-old *capza1b*^*ex5*^ homozygotes that were raised under anaesthetic conditions featured actin-positive aggregates at the peripheral ends of myofibres. (E) The length of rostral myomeres within 3-dpf-old *capza1b*^*ex5*^ homozygotes (84.0 ± 0.8 μm) was significantly shorter compared to myomeres within siblings (97.6 ± 0.7 μm) (n = 10 larvae with 1 myomere each, P < 0.001). (F) Electron-dense aggregates were not found in transmission electron micrographs of 3-dpf-old siblings. In contrast, electron-dense aggregates were found in *capza1b*^*ex5*^ homozygotes that were often characterised by a lattice structure (arrow), as depicted under higher magnification of the boxed area. Scale bar sizes are indicated.
(TIF)

S4 Fig Compound *capza1a*^*ex5*^;*capza1b*^*ex5*^ homozygotes feature aggregates with lattice structure. At 3 dpf, transmission electron micrographs of WT siblings show highly order sarcomeres. In contrast, electron-dense aggregates (arrowheads) were found in *capza1a*^*ex5*^;*capza1b*^*ex5*^ compound homozygotes that often featured a lattice structure.
(TIF)

S1 Data Raw data of values presented in graphs within figures. (XLSX)
