# Supplementary figures and images for: Genetic dissection of novel myopathy models reveals a role of CapZα and Leiomodin 3 during myofibril elongation
Source: PLoS Genet. 2022 Feb 11;18(2):e1010066. doi: 10.1371/journal.pgen.1010066 (PMC8870547; doi:10.1371/journal.pgen.1010066)

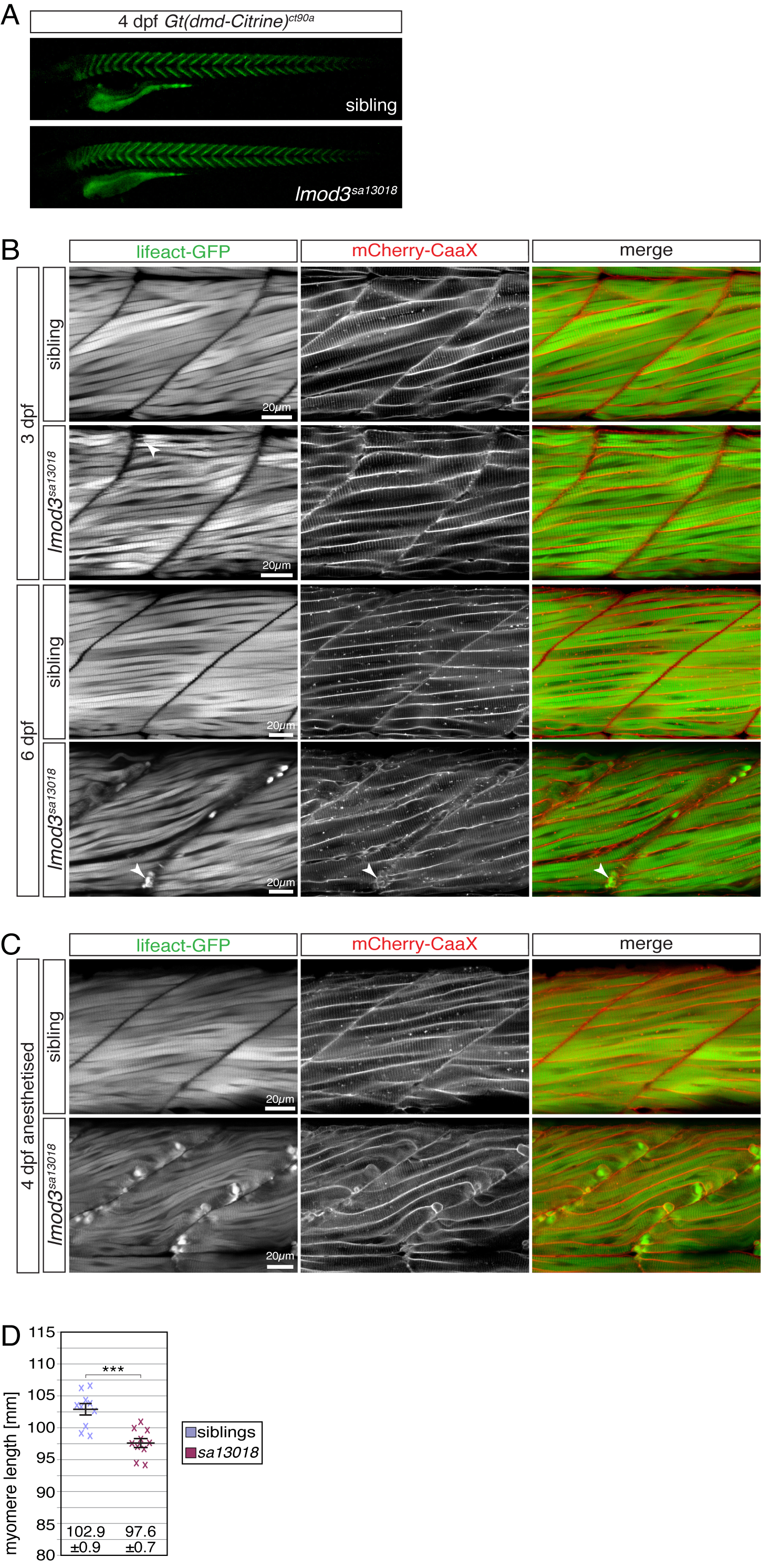

Supplement: S1 Fig — (A) At 4 dpf, the dmd-GFP fusion protein expressed by Gt(dmd-Citrine)ct90a localised at the myotendinous junction in siblings and lmod3sa13018 homozygotes. (B) Actin-positive aggregates were not detected in the background of Tg(acta1:lifeact-GFP) (green) and Tg(acta1:mCherryCaaX) (red) at 3dpf; neither in siblings nor lmod3sa13018 homozygotes. However, first signs of disconnected myofibril were apparent (arrowhead). At 6 dpf, aggregates marked by Lifeact-GFP were all localised in lmod3sa13018 at the peripheral end of myofibres. (C) In contrast to 4-dpf-old siblings, aggregates formed and localised at the peripheral end of myofibres also in lmod3sa13018 homozygotes that were raised under anaesthetising conditions to prevent force generation. (D) Whereas the length of rostral myomeres between vertical myosepta was 109 ± 0.9 μm in 4-dpf-old siblings, myomeres within lmod3sa13018 mutants were 97.6 ± 0.7 μm long and significantly shorter in comparison to their siblings (n = 10 larvae with 1 myomere each, P < 0.001). Scale bar sizes are 20 μm. (TIF) [file pgen.1010066.s001.tif]

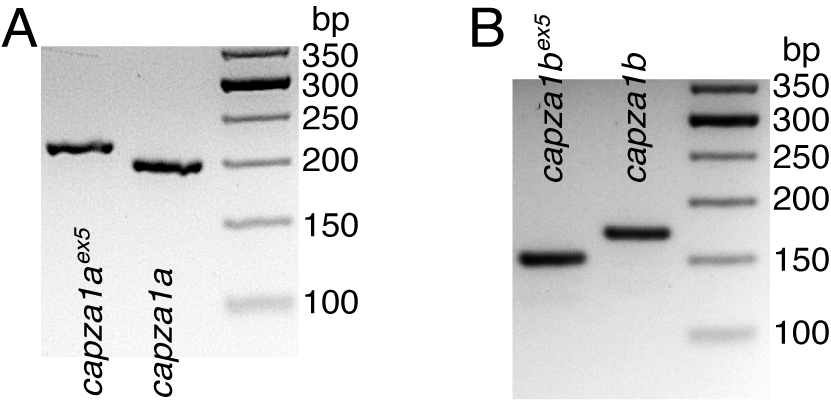

Supplement: S2 Fig — (A) RT-PCR using RNA isolated from 3-dpf-old capza1aex5 homozygotes generated a single 220-bp amplicon compared to the 200-bp amplicon generated with RNA obtained from wildtype siblings. The primers capza1a-2F (5’-acatggatcagttcacacctgc-) and capza1a-R (5’- tacagcatctctccacggcc-) were used to amplify capza1a cDNA. (B) Whereas the RT-PCR using RNA isolated from 3-dpf-old capza1bex5 homozygotes resulted in a 151-bp amplicon, a 173-bp amplicon was generated with RNA isolated from wildtype siblings. Amplicon sizes are indicated. PCR with capza1b cDNA was performed with capza1b-2F (5’-tggatggcagtgaggagtcg-) and capza1b-2R (5’-acaagcgtctctccaggacc-). (TIF) [file pgen.1010066.s002.tif]

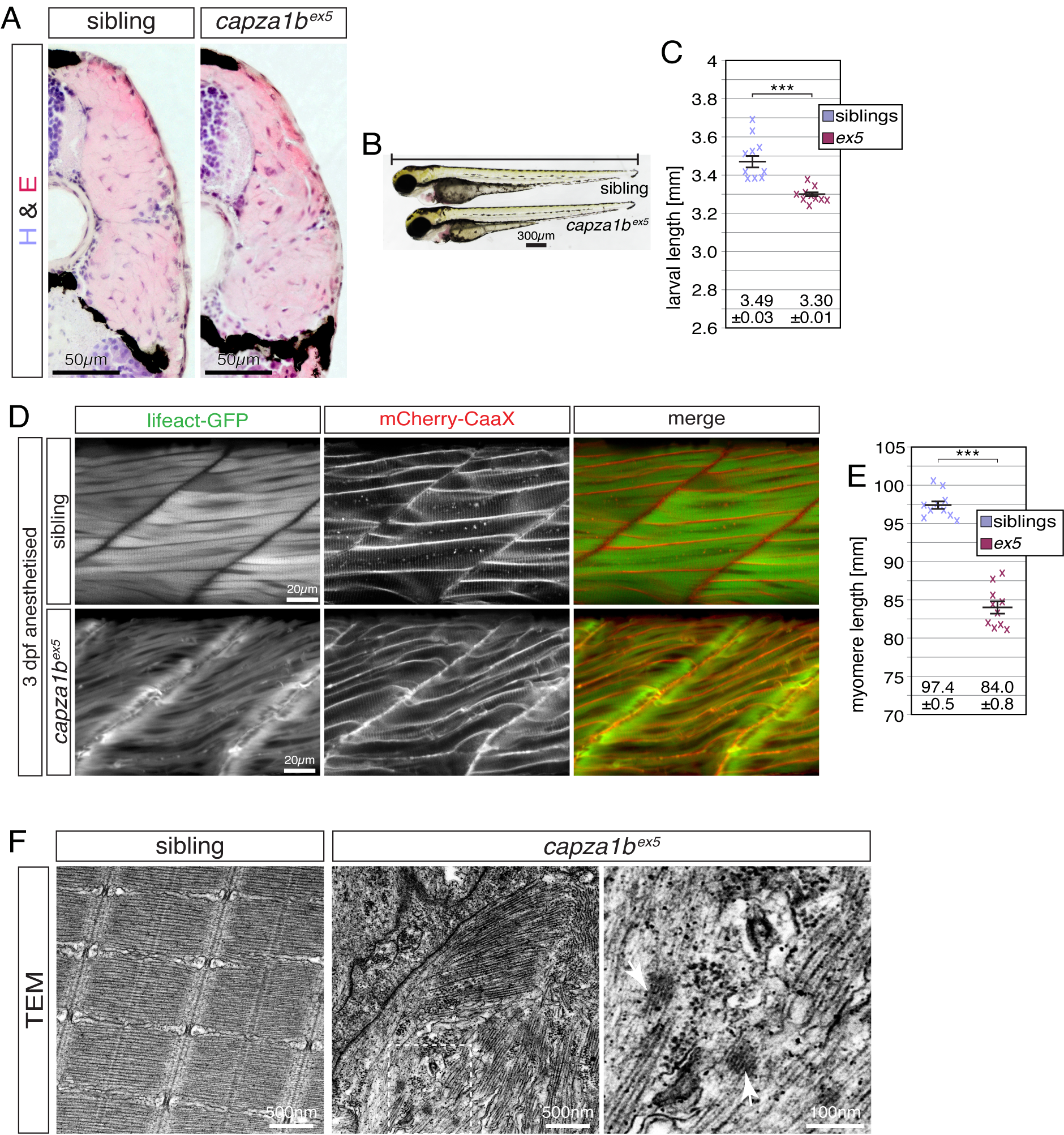

Supplement: S3 Fig — (A) At 6 dpf, signs of fibrosis or necrosis were absent on H&E-stained cross sections of capza1bex5 and siblings. (B) At 3 dpf, capza1bex5 homozygotes appeared shorter compared to their siblings. (C) Measurement of the body length excluding the caudal fin revealed that 3-dpf-old capza1bex5 homozygotes with a body length of 3.30 ± 0.01 mm were significantly shorter compared to their 3.49 ± 0.01 mm long siblings. Data are presented as mean ± SEM; n = 10 and *** P < 0.001 calculated by Student’s t test. (D) As depicted in the Tg(acta1:lifeact-GFP) and Tg(acta1:mCherry-CaaX) background, also 3-dpf-old capza1bex5 homozygotes that were raised under anaesthetic conditions featured actin-positive aggregates at the peripheral ends of myofibres. (E) The length of rostral myomeres within 3-dpf-old capza1bex5 homozygotes (84.0 ± 0.8 μm) was significantly shorter compared to myomeres within siblings (97.6 ± 0.7 μm) (n = 10 larvae with 1 myomere each, P < 0.001). (F) Electron-dense aggregates were not found in transmission electron micrographs of 3-dpf-old siblings. In contrast, electron-dense aggregates were found in capza1bex5 homozygotes that were often characterised by a lattice structure (arrow), as depicted under higher magnification of the boxed area. Scale bar sizes are indicated. (TIF) [file pgen.1010066.s003.tif]

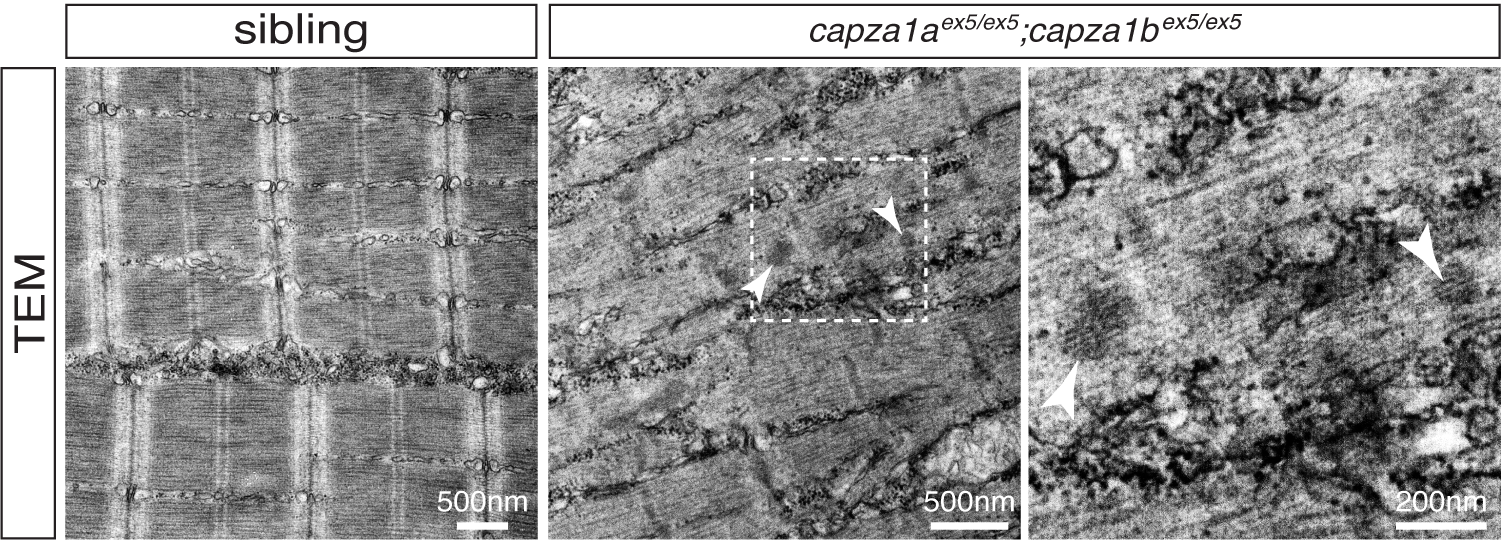

Supplement: S4 Fig — At 3 dpf, transmission electron micrographs of WT siblings show highly order sarcomeres. In contrast, electron-dense aggregates (arrowheads) were found in capza1aex5;capza1bex5 compound homozygotes that often featured a lattice structure. (TIF) [file pgen.1010066.s004.tif]
